# Extracellular Vesicles and Cancer Multidrug Resistance: Undesirable Intercellular Messengers?

**DOI:** 10.3390/life13081633

**Published:** 2023-07-27

**Authors:** María Bucci-Muñoz, Aldana Magalí Gola, Juan Pablo Rigalli, María Paula Ceballos, María Laura Ruiz

**Affiliations:** 1 Facultad de Ciencias Bioquímicas y Farmacéuticas (UNR), Instituto de Fisiología Experimental (CONICET), Rosario 2000, Argentina; bucci@ifise-conicet.gov.ar (M.B.-M.); gola@ifise-conicet.gov.ar (A.M.G.); ceballos@ifise-conicet.gov.ar (M.P.C.); 2 Department of Clinical Pharmacology and Pharmacoepidemiology, Heidelberg University Hospital, Im Neuenheimer Feld 410, 69120 Heidelberg, Germany; jprigalli@gmail.com

**Keywords:** ABC transporters, drug resistance, extracellular vesicles, P-glycoprotein, breast cancer resistance protein, multidrug resistance-associated protein

## Abstract

Cancer multidrug resistance (MDR) is one of the main mechanisms contributing to therapy failure and mortality. Overexpression of drug transporters of the ABC family (ATP-binding cassette) is a major cause of MDR. Extracellular vesicles (EVs) are nanoparticles released by most cells of the organism involved in cell–cell communication. Their cargo mainly comprises, proteins, nucleic acids, and lipids, which are transferred from a donor cell to a target cell and lead to phenotypical changes. In this article, we review the scientific evidence addressing the regulation of ABC transporters by EV-mediated cell–cell communication. MDR transfer from drug-resistant to drug-sensitive cells has been identified in several tumor entities. This was attributed, in some cases, to the direct shuttle of transporter molecules or its coding mRNA between cells. Also, EV-mediated transport of regulatory proteins (e.g., transcription factors) and noncoding RNAs have been indicated to induce MDR. Conversely, the transfer of a drug-sensitive phenotype via EVs has also been reported. Additionally, interactions between non-tumor cells and the tumor cells with an impact on MDR are presented. Finally, we highlight uninvestigated aspects and possible approaches to exploiting this knowledge toward the identification of druggable processes and molecules and, ultimately, the development of novel therapeutic strategies.

## 1. Introduction

### 1.1. Multidrug Resistance (MDR)

Around the world, cancer ranks as a leading cause of death and represents a significant barrier to raising life expectancy [1]. According to the estimates performed by GLOBOCAN in 2020, the total number of new cancer cases and deaths was recorded as 19.3 million and almost 10.0 million worldwide, respectively [2]. The most commonly diagnosed cancer is female breast cancer (2.3 million new cases; 11.7%), followed by lung (11.4%), colorectal (10.0%), prostate (7.3%), and stomach (5.6%) cancers. Regarding mortality, lung cancer is the leading cause of cancer death (1.8 million deaths; 18%), followed by colorectal (9.4%), liver (8.3%), stomach (7.7%), and female breast (6.9%) cancers. The global cancer burden is expected to increase by 47% by the year 2040, reaching 28.4 million cases [2].

In order to improve the lifespan of cancer patients and to eradicate cancer, several therapeutic strategies have been explored, from highly invasive surgical resections to radiation-induced eradication and diverse therapeutic approaches like chemotherapy, targeted therapy, and, more recently, immunotherapy [3,4]. However, several factors like the age of diagnosis, treatment time, and stage of cancer, among others, affect the efficacy of treatment. In addition, most drugs present serious adverse effects due to the lack of target selectivity [3]. More importantly, resistance to already available drugs, which allows cancer to proliferate in the presence of a chemotherapeutic agent, has emerged as a major obstacle for cancer therapies, being associated with more than 90% of cases of treatment failure [3,5]. This mechanism, known as multidrug resistance (MDR) or chemoresistance, consists of the resistance of tumor cells to different drugs with multiple chemical structures, multiple mechanisms of action, and multiple targets and, ultimately, renders the cancer cell ineffective in achieving the therapeutically required intracellular drug concentration. Indeed, cancer cells present cross-resistance to a broad spectrum of structurally unrelated chemotherapeutic agents and this mechanism of MDR can be intrinsic or acquired. Intrinsic resistance refers to a complete failure of response to initial antitumor therapy in patients, i.e., cancer cells are already resistant before treatment. In acquired resistance, on the other hand, cancer cells become resistant during treatment and therefore the cancer initially responds to therapy but then relapses or progresses after a period of time. Most tumors in clinical settings become resistant owing to a combination of both types of resistance [3,5]. Unfortunately, the mechanisms implicated are not yet fully understood, and MDR is the major cause of cancer recurrence and metastasis, and it is related to a longer period of hospitalization, increased medical costs, and high mortality [4].

Frequently, when tumor cells face a new stress, such as a chemotherapeutic agent, they may adapt by acquiring a series of mutations, become resistant and, in this way, survive therapy [6]. MDR is a multifactorial phenomenon, with diverse resistance mechanisms to the same chemotherapeutic agent acting on the same cancer cell. In this way, it is very complex to inhibit MDR because when one resistance mechanism is blocked, cancer cells adapt and develop MDR through other mechanisms. The multiple mechanisms, which are associated with a complex process of multiple genes, factors, pathways, and multiple steps, may include host factors, tumor factors, as well as tumor–host interactions [6,7]; microbiomes may also play a role in cancer drug resistance [8].

Host factors include the genetic variants between patients that affect the efficacy of therapy, like single nucleotide variants, insertions, deletions, repeats, chromosomal rearrangement, and copy number variations [3,6]. Also, cancer patients are very susceptible to drug–drug interactions because they commonly receive complementary medications during therapy. Coadministered drugs may change the efficacy and/or toxicity of the chemotherapeutic agent by modifying its absorption, distribution, metabolism, or excretion (pharmacokinetic) and can result in synergistic, antagonistic, or additive responses (pharmacodynamics) [7].

Several tumor factors have been described and, additionally, the function of some of the proteins involved in these mechanisms could be affected by the presence of genetic variants between individuals (host factors) [4,7,9]. The predominant mechanism contributing to MDR results from the increased drug efflux by ATP-dependent pumps located on the surface of cancer cells. These transmembrane transporters belong to the ATP-binding cassette (ABC) superfamily and their expression is frequently upregulated in tumor cells [3,4,5]. Also, drug efflux can be mediated by extracellular vesicles (EVs) with the ability to sequester chemotherapeutic drugs, extruding them from cells once released from tumor cells. ABC transporters have been found in the cargo of EVs in an inverted orientation, contributing to the influx of drugs into the EVs [7]. Similarly, another mechanism is the reduced uptake of the drug due to changes in the expression and/or activity of transporters involved in the transport of the chemotherapeutic agent inside the cancer cell or by the reduction in drug diffusion by a barrier to drug permeability established in these cells. The sequestration of chemotherapeutic agents in intracellular vesicles and compartments away from the cellular target, like the lysosomal compartmentalization of drugs, also contributes to MDR. In addition, alterations in the biotransformation or metabolism of the drug by modifications in the expression and/or activity of drug-metabolizing enzymes could lead to impaired prodrug activation or increased proportion of inactive metabolites, reducing the intracellular concentration of the active drug [3,7,9]. Since drugs need to be active and reach their targets at an adequate concentration to achieve therapeutic efficacy, the above mechanisms directly influence the outcome of therapy by decreasing the available intracellular drug concentration [9].

The plethora of tumor factors also involve mutations or changes in the expression levels of target genes of the antitumor drugs that prevent an efficient drug–target interaction in proteins involved in survival and cell death (that allow cancer cells to survive) and in proteins related to DNA repair mechanisms (which enable cancer cells to repair the DNA damaged by chemotherapeutic agents and avoid cellular death while promoting genomic instability and mutations) [3,4,9]. Epigenetic alterations, such as DNA methylation and histone and chromatin modifications, are another tumor factor that can change the expression of numerous MDR genes, like those coding for ABC transporters. Other tumor factors are the deregulation of microRNA (miRNAs) implicated in the regulation of MDR proteins, modifications in the epithelial–mesenchymal transition (which leads to a dedifferentiation of epithelial cells to a mesenchymal phenotype), and cancer stemness related to cancer stem cells. Finally, the heterogeneity within an individual tumor or between tumors (interindividual) is another tumor factor that provides genetic and epigenetic diversity, allowing for therapy-induced expansion of preexisting drug-resistant cancer cell clones [4,6,9].

Tumor–host interactions are mainly linked to the tumor microenvironment (TME), a highly complex ecosystem consisting of extracellular matrix, soluble factors, and tumor and stromal cells, which imposes a selective pressure that drives tumor progression [3,7]. This selective pressure includes hypoxia, extracellular acidosis, nutrient deprivation, immune surveillance, and unorganized leaky vasculature, which influence MDR by altering the distribution of drugs inside tumors and also by affecting their cellular uptake. In addition, some of these characteristics of the TME modify the expression of MDR proteins, such as the ABC transporters. In addition to the aforementioned MDR mechanisms, other specific ones linked to the immune cells infiltrating the TME have also been described [4,10]. Additionally, extracellular vesicles (EVs) might contribute to the modulation of TME and to the dissemination of MDR by transferring MDR-associated proteins between tumor and stromal cells and between drug-resistant and drug-sensitive tumor cells [7].

The TME does not only bring complexity to the MDR phenomenon due to its diverse effects on chemoresistance, but also to the effect of microbes that can translocate and reside in tumor niches. The microbes are able to inactivate anticancer drugs via biotransformation or by activating oncogenic pathways inducing chemoresistance. In patients with colorectal cancer, an important intratumoral bacteria, *Fusobacterium nucleatum*, has been shown to promote the development of oxaliplatin resistance during treatment by activating the innate immune system and inducing autophagy [8]. Increased levels of the oral pathogens *Aggregatibacter actinomycetemcomitans* and *Porphyromonas gingivalis* were observed in pancreatic cancer patients, which promoted the expression of cytidine deaminase and impacted the occurrence of chemoresistance. In the mouse colorectal cancer model CT26, intratumoral administration of *E. coli* not only affected the activity and concentration of gemcitabine at the tumor site, but also affected the development of drug resistance [11,12]. Intratumoral gammaproteobacteria with long isoform of cytidine deaminase can inactivate gemcitabine, leading to chemoresistance [12,13]. Intratumor-residing gut microbiota could modulate chemokine levels and affect CD8+ T cell infiltration, consequently influencing patient survival in cutaneous melanoma. Manipulating the intratumor gut microbiome may benefit patient outcomes for those with immunotherapy [14].

**Figure 1:**
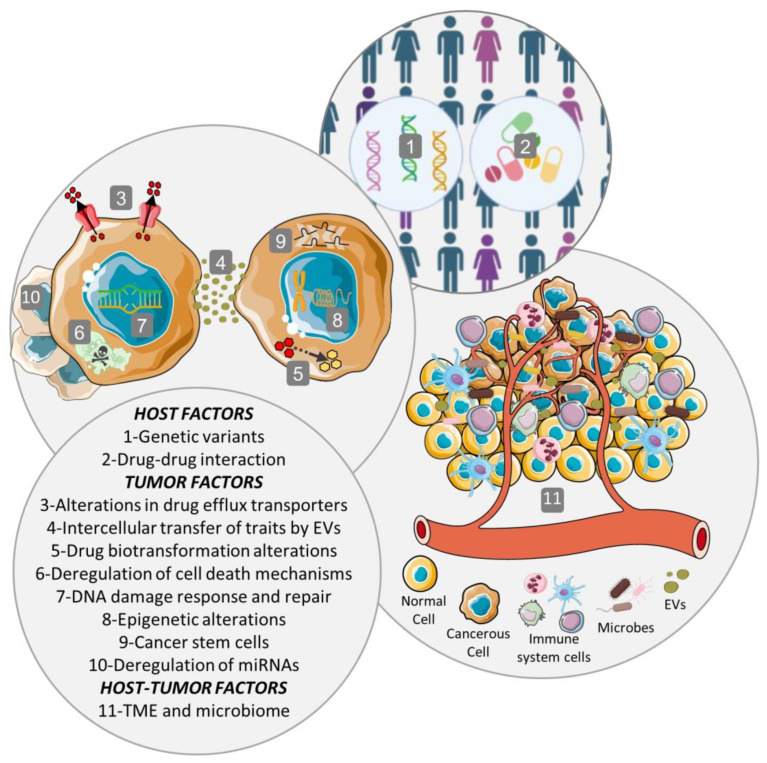
Multifactorial phenomenon of MDR. The multiple mechanisms, which are associated with a complex process of multiple genes, factors, pathways, and multiple steps, may include host factors, tumor factors, as well as tumor–host interactions. Host factors include host genetic variants and drug–drug interactions. Tumor factors comprise alterations in intracellular drug concentration originated by modifications in drug efflux or influx transporters or by extracellular vesicles (EVs), alterations in the biotransformation or metabolism of the drug, deregulation of cell death mechanisms, DNA damage response and repair, epigenetic alterations, cancer stem cells, and deregulation of microRNAs (miRNAs). Among the tumor–host factors are the tumor microenvironment (TME), the selective pressures and tumor evolution, intercellular transfer of traits by extracellular vesicles (EVs), and tumor microbiome. This figure was partially generated using Servier Medical Art, provided by Servier (Suresnes, France), licensed under a Creative Commons Attribution 3.0 unported license.

All mechanisms mentioned in this section are illustrated in Figure 1.

### 1.2. ABC Transporters

The ABC superfamily is identified by the presence of a group of highly conserved motifs. Structurally, all ABC transporters consist of at least two transmembrane domains (TMDs), which are inserted in the lipid bilayer, and two nucleotide-binding domains (NBDs) located in the cytoplasm [15]. Some exceptions to this rule are transporters whose sequence spans a single NBD and a single TMD. In the latter case, the transporter needs to be dimerized to obtain full functionality [16]. TMDs have a conserved three-dimensional structure, consisting of a core of six transmembrane α-helices per TMD. These domains are hydrophobic and structurally diverse; they recognize and translocate various substrates through conformational changes. The variability of the sequences and structures of the different TMDs is reflected in the chemical diversity of the substrates they transport [17]. NBDs are domains consisting of a highly conserved ABC motif, which is responsible for binding and hydrolyzing ATP, thus providing the energy for the transport of physiological and xenobiotic substrates [18]. In addition to the ABC motif, NBDs also contain other motifs and typical conformations that characterize them, such as the Walker A and B motifs [19].

ABC transporters can be classified based on the structure and sequence of the NBDs (also known as ABC domains) [20]. There are several synonyms for members of this family. Currently, the systematic nomenclature used is based on the organization of the domains and homologies of their amino acids. In this nomenclature, ABC transporters are divided into seven distinct subfamilies: ABCA, ABCB, ABCC, ABCD, ABCE, ABCF, and ABCG [21,22].

Regulation of ABC transporters can be mediated by changes in the number or activity of transporter proteins. These modifications can occur in the long term (i.e., in terms of days) when it comes to regulation of transporter gene expression, caused by ligands such as hormones or drugs, which activate nuclear receptors involved in the transcription process, or due to changes in their rate of degradation (half-life of the transporter), or in the short term (substrate availability, phosphorylation–dephosphorylation of transporters, covalent modifications, insertion regulated by exocytosis or endocytic withdrawal) [23].

Within the large family of ABC transporters, ABCB1 (P-glycoprotein/MDR1), as well as ABCC1 (MRP1), ABCC2 (MRP2), ABCC3 (MRP3), and ABCG2 (BCRP) are the transporters mostly involved in the development of MDR in different types of tumors. For the sake of clarity, we refer here to the transporters as P-gp, MRP, or BCRP, as this is the denomination most frequently used in the original articles.

#### 1.2.1. P-Glycoprotein/ABCB1/MDR1

The first member of the ABC transporter superfamily, P-gp (ABCB1/MDR1), was identified in 1976 by Juliano and Ling as a 170 kDa membrane glycoprotein. P-gp was found overexpressed in colchicine-resistant ovarian cell lines and reduced the permeability to this drug [24]. After that, it became the most studied ABC transporter and the one with the broadest substrate specificity [25], as it transports a large variety of molecules with different chemical structures and molecular weights [26]. There is a positive correlation between *MDR1* expression and high risk of disease progression in ovarian cancer patients, as was demonstrated previously [27]. Additionally, an inverse relationship between paclitaxel response and P-gp expression has been reported [28].

In epithelial polarized cells, P-gp localizes to the apical membrane of renal proximal tubular cells, cells from placenta, hepatocytes, enterocytes, adrenal cells, and blood–brain barrier cells, where it functions to protect against xenobiotics and cellular toxicants [29,30,31,32,33]. P-gp is also expressed on CD34+ hematopoietic progenitor cells, natural killer cells, and CD8+ T cells [34]. P-gp expression was analyzed in 60 cell lines from a wide range of tumors and detected in 39 of them using quantitative real-time PCR. The highest levels of P-gp were found in renal and colon carcinomas [35]. P-gp is frequently also detected in cancer stem-like cells.

Lee et al., using a CellTiter-Glo viability assay, tested a total of 10,804 library compounds, most of which have known mechanisms of action. Of the tested compounds, a total of 90 putative P-gp substrates were identified, including 55 newly identified compounds [36]. The substrates transported by P-gp include endogenous compounds such as steroid hormones, lipids, peptides, and small cytokines [20,21,37]; short-chain phospholipids, sphingolipids, organic cations, carbohydrates, amino acids, and macromolecules such as polysaccharides and proteins [38,39]. It also transports compounds of exogenous origin that are toxic to the cell, for example, some herbicides [40]. The ability of P-gp to pump xenobiotics becomes disadvantageous when it interferes with drug accumulation in target tissues. P-gp transports a very broad spectrum of drugs including digoxin, doxorubicin, vinblastine, paclitaxel, rifampicin, clarithromycin, and atorvastatin [41].

*MDR1* (i.e, gene codifying P-gp) expression can be induced by a variety of environmental stimuli, such as ultraviolet radiation [42]. *MDR1* expression is also induced by compounds such as sodium butyrate, retinoic acid, phorbol esters, and even many chemotherapeutic drugs [42]. The latter are able to induce specific epigenetic modifications at the MDR1 locus, together with the induction of P-gp, at protein level, through transcriptional activation. Such a mechanism is dependent on the methylation status of the MDR1 promoter [43]. Binding of specific transcription factors to DNA sequences, located distal or proximal to the *MDR1* promoter region, as well as methylation/demethylation, modulate *MDR1* transcriptional expression [44]. Hypermethylation of the *MDR1* promoter has been found to be associated with activation of transcription-blocking mechanisms, which are independent of histone deacetylase. Methylation and acetylation of lysine residues in the N-terminal fragments of histones are important mechanisms of *MDR1* expression regulation [38]. Scotto and colleagues (2003) have shown that the previously mentioned stimuli, inducers of *MDR1* expression, converge on a region of the promoter of this gene, which they called the “enhancer”. This promoter region, like that of most genes that do not have a “TATA box” in their sequence, includes an inverted “CCAAT box” (which interacts, for example, with the trimeric transcription factor NF-Y) and a GC-rich element (which interacts with members of the Sp family of transcription factors, such as Sp1) [44]. In addition to NF-Y and Sp1, P-gp expression is also transcriptionally regulated by nuclear receptors that act as xenosensors. Among them we can name the pregnane X receptor (PXR) and the constitutive androstane receptor (CAR), which induce *MDR1* transcription [45,46]. Under oxidative stress conditions, there is evidence that the nuclear factor erythroid 2-related factor 2 (Nrf2), which is activated to protect the cell from free radical damage, is able to modulate *MDR1* expression [47,48]. On the other hand, several steroid hormones can also modulate *MDR1* through the estrogen receptor (ER, estrogen receptor) [49,50]. *MDR1* promoter activity can also be stimulated by the tumor suppressor protein p53 and the Ras oncogene product proteins Ras, c-Raf, and c-Raf kinase [5]. P-gp expression can also be regulated at the posttranscriptional level. MiRNAs are a class of short, noncoding RNA molecules that regulate gene expression. Through bioinformatics analysis, several miRNAs were found to bind to the 3’ end of the untranslated regions (UTRs) of the MDR1 mRNA [5]. In fact, mRNA UTRs can play an essential role in regulating protein synthesis and the half-life of different mRNAs in the cell. For example, the miRNAs miR-223 and miR-145 decrease P-gp expression by direct action on the 3’-UTR of *MDR1* mRNA, binding to it and preventing translation. miRNA-27a and miRNA-138, instead, regulate P-gp expression at the level of transcription [51]. In addition to miRNAs, several drugs, such as colchicine, doxorubicin, colcemid, vinblastine, cytarabine, and ivermectin, regulate P-gp expression by stabilizing its mRNA [5,38,52,53].

#### 1.2.2. MRPs/ABCCs

The ABCC/MRP subfamily consists of at least nine homologs expressed in humans, named MRP1 to MRP9 [54,55,56,57]. They exhibit a conserved structure but heterogeneity in terms of substrate specificity, localization, and function. The first member of this subfamily, MRP1, was discovered by Cole in 1992, in a lung cancer cell line resistant to doxorubicin without overexpression of P-gp [58].

MRP1 is expressed in normal tissues such as the kidney, intestine, blood–brain barrier, lung, testis, and mononuclear cells [59]. Normal liver expresses MRP1 at very low levels [60]. However, an isoform of MRP1, located in the canalicular membrane, was later discovered in the liver and named MRP2 [61]. Its expression is not restricted only to the liver, but is also present in the kidney, small intestine, and blood–brain barrier [23]. Subsequently, a basolateral isoform called MRP3/ABCC3 [62] was identified in the sinusoidal membrane of hepatocytes, where it mediates the vectorial transport of substrates from the hepatocyte to the sinusoidal blood [62,63,64]. It was also detected in the kidney, intestine, pancreas, lung, gallbladder [65,66], as well as in different tumors where its expression was associated with increased chemoresistance [67,68]. MRP1 is capable of transporting chemotherapeutic agents such as anthracyclines, camptothecin analogues, and vinca alkaloids. MRP1 is overexpressed in lung, breast, prostate, and ovarian cancer as well as in gastrointestinal carcinoma, melanoma, and leukemia [69]. MRP2 is capable of transporting conjugated compounds like acetaminophen glucuronide or ethynyl estradiol glucuronide [23] and also unconjugated drugs such as methotrexate, ritonavir, saquinavir, vinblastine, sorafenib, and cisplatin [23,70]. MRP3 is able to transport and thus participate in methotrexate and etoposide resistance [67,68]. MRP3 has also been shown to participate in resistance to sorafenib in HCC [71]. Additionally, MRP4 and -5 can also confer resistance against some chemotherapeutic drugs and are able to transport certain organic anions [72,73,74].

The expression of MRPs as well as P-gp is regulated at different levels. Transcriptional modulation takes place through the activation of nuclear receptors by several endo- or xenobiotics or by different oncoproteins and transcription factors that bind to the promoter regions of MRPs. This includes c-jun/junD complexes [75]; MYC and MYCN [76]; SOX2 [77]; ATF4 [78]; and Nrf2 [79]. Regarding nuclear receptors, PXR, CAR, and the farnesoid X receptor (FXR, NR1H4) have been shown to mediate MRP2 upregulation by several drugs and other endo- and xenobiotics [80,81,82,83]. Moreover, the MRP2 promoter harbors glucocorticoid response elements (GRE) and antioxidant response elements (ARE) mediating the effect of glucocorticoids via the glucocorticoid receptor on the transporter expression [77]. MRP2 and MRP4 were shown to be positively regulated by Nrf2, as was described for MRP1 [79,84,85,86], whereas MRP1 is negatively regulated by the peroxisome proliferator-activated receptor α (PPARα, *NR1C1*) in the small intestine [87].

MRPs can undergo translational regulation in which a change in the protein expression takes place without a concomitant change in the mRNA synthesis rate, as described in ethynyl estradiol- and pregnancy-associated cholestasis [88,89]. Among miRNAs that modulate MRPs are miRNA- 326 and miR-145, which downregulate MRP1, miRNA-379, and miR-490-3p, which modulate MRP2 [90,91] and miRNA-149, which modulates MRP3 [92].

#### 1.2.3. BCRP/ABCG2

In 1998, Doyle et al. described an ATP-dependent reduction in the intracellular accumulation of anthracyclines in MCF7 cells and resistance to doxorubicin, without overexpression of known multidrug resistance transporters such as P-gp or MRPs [93]. This new ABC transporter was named BCRP (breast cancer-resistant protein/ABCG2). The human BCRP gene is located on chromosome 4q22 4q21, and encodes a unique 72 kDa peptide consisting of only one NBD and one TMD, and is therefore referred to as a “half transporter” [94]. BCRP is also known as MXR, because it mediates the efflux of mitoxantrone and is therefore responsible for resistance to this frequently used chemotherapeutic compound [95] in tumor cells. In addition, this transporter is associated with drug resistance in breast cancer [96]. BCRP is expressed in numerous organs/tissues, including the placenta, liver, gastrointestinal tract, kidney, prostate, breast, adrenal gland, and the luminal surface of endothelial cells of human brain microvessels. BCRP protects normal cells from xenobiotic toxicity in order to maintain physiological homeostasis [97]. Overexpression of BCRP renders cancer cells resistant to multiple drugs such as mitoxantrone, topotecan, and methotrexate and is associated with poor response to chemotherapy in leukemia and breast cancer patients [69]. In addition, a negative correlation between BCRP expression and prognosis in breast cancer has been described [98].

Moreover, two epigenetic mechanisms appear to cooperatively regulate BCRP expression in the drug-resistant phenotype: histone hyperacetylation of the BCRP promoter [99] and CpG island demethylation [100]. Regarding posttranscriptional regulation, different miRNAs (e.g., miR-519c, miR-520h, miR-328, miR-181a, and miR-487a) are involved in the downregulation of BCRP [101,102].

### 1.3. Extracellular Vesicles

Extracellular vesicles (EVs) are particles released by most cells of the organism to the extracellular space. According to their mechanisms of biogenesis and release, EVs are classified into exosomes, microvesicles (MVs, also known as ectosomes or microparticles), and apoptotic bodies. Exosomes originate from endosomal compartments. MVs originate through outward budding of the plasma membrane. Apoptotic bodies are released during programmed cell death. In terms of their size, most exosomes have a diameter between 40 and 100 nm, MV diameters usually fall between 200 and 1000 nm, and apoptotic bodies are larger than 1 µm. The EV cargo comprises proteins, nucleic acids (mRNAs, miRNAs, other noncoding RNAs), carbohydrates, and lipids. Although size distributions partially allow for distinguishing between exosomes, MVs, and apoptotic bodies, from smaller to larger particle sizes, particle distributions also overlap [103]. In addition, the current EV isolation methods (e.g., ultracentrifugation, size exclusion chromatography, precipitation, ultrafiltration, affinity capture) are mostly based on physicochemical properties common to more than one type of EV. Therefore, the isolation of a specific type of vesicles remains a difficult, mostly unaccomplished task. In agreement with the recommendations of the International Society for Extracellular Vesicles (ISEV) [104], we use the term EVs throughout the manuscript, irrespective of the nomenclature used in the original sources. A clear mention to a specific EV type (e.g., MVs) is made only when the isolation methods used clearly allow for the isolation of that type of EV with negligible contamination by other types of vesicle.

Functionally, EVs have been demonstrated to play a major role in cell–cell communication in health [105] and disease [106]. EV participation in the pathogenesis of several disorders has been demonstrated. Among them, EVs have been reported to play a major role in cancer cell proliferation, metastasis, and immune evasion [106]. Several studies have also addressed the role of EVs in relation with cancer multidrug resistance using different models in vitro and in vivo.

## 2. EVs as Mediators of Multidrug Resistance

The most frequent mechanism of EV-mediated drug resistance consists of the release of EVs by donor cells with a MDR phenotype, followed by uptake by drug-sensitive cells (i.e., target cells), which, in response, acquire a drug-resistant phenotype. Furthermore, donor cells may not necessarily be tumor- or multidrug-resistant cells. For instance, canine natural killer cell-derived EVs injected to a mouse xenograft bearing canine mammary tumors resulted in an increase in P-gp expression in the tumor cells. Although the functional relevance of P-gp upregulation was not investigated, this study clearly highlights the wide spectrum of EV-mediated cell–cell interactions with a potential impact on transporter modulation and, most likely, drug resistance [107].

Cell–cell communication via EVs is strongly determined by the cargo transported between donor and target cells. Furthermore, cargo-independent features may also influence the transfer of information. For instance, a study with drug-sensitive and multidrug-resistant non-small cell lung cancer (NCI-H460) and chronic myeloid leukemia (K562) cells identified an increase in EV release by drug-resistant cells. This observation could be attributed to the upregulation of the Rab GTPases Rab5 and Rab11, and Rab 27 in drug-resistant NCI-H460 and K562 cells, respectively, compared to their drug-sensitive counterparts. In addition, drug-sensitive cells exhibited an increase in EV uptake, independent of the phenotype of the donor cells. These features, although not directly impacting the EV cargo, may also favor the vesicle-mediated cell–cell communication process [108]. Another study investigated the size distribution of EVs released by the same cell types. Results pointed to a predominant release of larger EVs by drug-resistant NCI-H460 and K562 cells. On the contrary, drug-sensitive cells release a higher proportion of exosomes, as determined based on the EV size and the expression of exosome markers [109].

**Figure 2:**
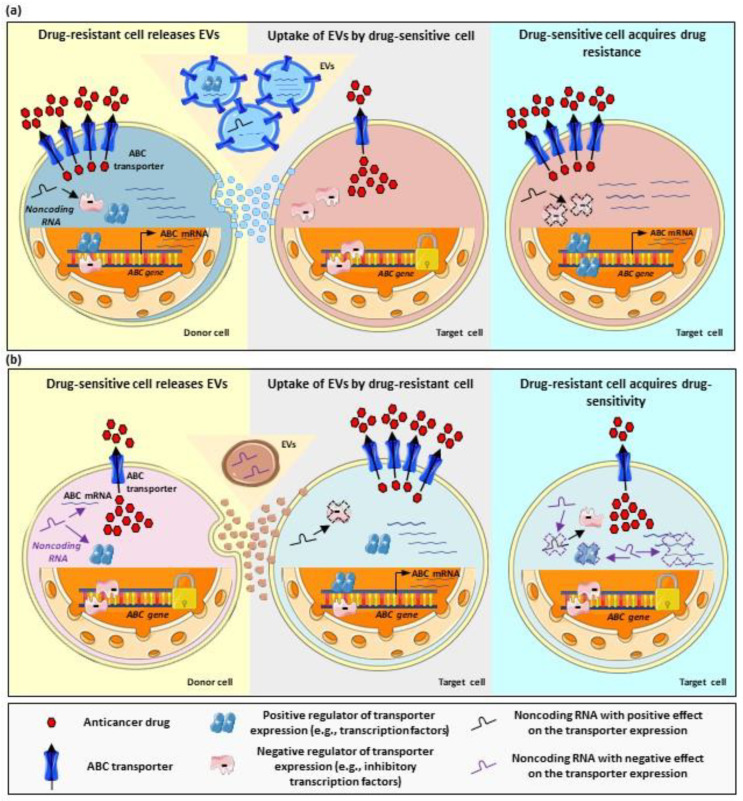
Regulation of multidrug resistance by EV-mediated cell–cell communication. (**a**) Stimulation of drug resistance by EVs. Drug-resistant cells (donor cells) with overexpression of ABC transporters exhibit lower accumulation of anticancer drugs (left panel). These cells can release EVs carrying, for example, ABC transporters, their coding mRNAs, noncoding RNAs, and proteins with a positive regulatory function. Once taken up by target cells (originally drug-sensitive cells, central panel), the components of the EV cargo result in the upregulation of ABC transporters. This takes place, for example, by direct shuttling of transporter molecules or their codifying mRNAs; but also by shuttling of regulatory noncoding RNAs, which may inhibit the function of negative regulators; or by shuttling of positive regulators (e.g., transcription factors). Ultimately, the stimulation of drug resistance in the target cell takes place (right panel). (**b**) Inhibition of drug resistance by EVs. Drug-sensitive cells (donor cells, left panel) exhibit lower transporter expression and, therefore, increased drug accumulation. These cells usually overexpress negative regulators of the transporter expression and the function of positive regulators may be inhibited (for example, by noncoding RNAs). These cells release EVs, which can be taken up by target cells, originally drug-resistant (central panel), with transporter overexpression. The transfer of the EV cargo to target cells results in transporter downregulation, for example, by interaction of noncoding RNAs with the transporter mRNA and, therefore, inhibition of the translation; by inhibition of positive regulatory factors; or by activation of negative regulatory factors. Arrows indicate situations of up-regulation, down-regulation or inhibition. This Figure was partially generated using Servier Medical Art, provided by Servier, licensed under a Creative Commons Attribution 3.0 unported license.

A similar study, using drug-sensitive and drug-resistant cervical cancer cells, found higher P-gp expression in EVs released by the resistant clones. However, incubation of nontumoral cells (i.e., immortalized human fibroblasts) with EVs did not result in a transfer of the transporter or in the stimulation of other malignant features (i.e., migration) in target cells [110]. These findings point to additional cell-specific mechanisms regulating and influencing the directional transfer of information between different cell types. Thus, although the actual cargo of the EVs is the main factor determining the effect in terms of multidrug resistance, the identity as well as structural and functional features of donor and target cells may play a relevant role in the transfer of the drug-resistant phenotype as well. In the following sections, we discuss different cases of EV-mediated multidrug resistance based on the role played by vesicles and bioactive cargo. The most common mechanisms are also schematized in Figure 2. In addition, we address the potential of EVs as a source of biomarkers of drug-resistance.

### 2.1. EV-Mediated Shuttle of ABC Transporters

One of the most frequently described mechanisms of EV-mediated drug resistance is the direct transfer of drug transporters or its coding mRNA from a donor, in general, drug-resistant cell to a target, mostly, drug-sensitive cell. In this regard, using a coculture system, Pasquier et al. demonstrated the transfer of P-gp from drug-resistant to drug-sensitive MCF7 cells via EVs. As a result of this interaction, target cells acquired doxorubicin resistance. The authors also demonstrated higher P-gp levels in the vesicles derived from resistant cells compared to the vesicles obtained from drug-sensitive cells. Since both cell types were separated by a filter with a 0.4 μm pore size, EVs in the lower size range are likely to be involved in the transfer of resistance. Interestingly, the time dependence of the transfer was also investigated. Increased P-gp protein expression in target cells was already observed at 4 h of exposure to the EVs. Peak expression was registered at 12 h of exposure and a return to basal levels was observed from 36 h of exposure in advance. Additionally, an increase in P-gp activity in target cells was observed. Here, the kinetics of the effect exhibited a similar pattern, with the highest increase in transporter activity observed at 4 h of exposure and a return to basal levels at 24 h of exposure to the EVs [111]. Using a similar experimental setting, Wang et al. demonstrated the upregulation of P-gp and MRP1 mRNA in sensitive MCF7 cells treated with EVs from a doxorubicin-resistant MCF7 clone. As expected, treatment with EVs also stimulated resistance to doxorubicin in the sensitive clone. Higher P-gp protein levels in EVs from the resistant clone were observed. However, no data on the mRNA expression in the vesicles were obtained. Therefore, it is still unclear whether the mRNA upregulation mediated by EV treatment is a mere result of messenger transfer via EVs or whether it results from the transfer of other regulatory proteins or RNAs. Interestingly, in this study, the authors demonstrated an inhibitory effect of psoralen, a furanocoumarin, on EV release by the doxorubicin-resistant cells, thus suggesting a potential compound able to counteract the transfer of drug resistance [112]. Similar findings were described in another study using docetaxel-resistant MCF7 cells due to P-gp overexpression. As expected, EVs derived from these cells exhibited a higher expression of P-gp compared to those from drug-sensitive MCF7 cells and, once added to the latter, resulted in an increase in P-gp protein levels and resistance to docetaxel in target cells [113].

Another study using MCF7 and CEM (human acute lymphoblastic leukemia) cells investigated P-gp in MVs (300–600 nm) released by drug-resistant clones of both cell types. Addition of these MVs to drug-sensitive clones of the respective cell line resulted in significant P-gp upregulation at the mRNA level. Unexpectedly, a simultaneous decrease in MRP1 mRNA expression was observed in CEM cells treated with MVs from the resistant clone. The authors postulated that miR-326, which negatively regulates MRP1 mRNA expression and is carried in EVs together with transporter messengers, was responsible for MRP1 downregulation. These findings point to a complex picture of EV-mediated transfer of ABC transporters, where specific transporters can be up- or downregulated based on the cargo of the EVs. Concomitantly, increased or decreased resistance to specific chemotherapeutic agents, could be expected [114]. A further study from the same group analyzed, in addition, the cell specificity of transporter transfer via MVs. Interestingly, the nature of the donor cells was reported to play a key role. In fact, MVs derived from drug-resistant CEM cells transferred P-gp and MRP1 to both cancerous and noncancerous cells (i.e., human mammary basal epithelial cells, human osteoblasts, and human urothelial cells) while, on the contrary, MVs from drug-resistant MCF7 cells only transferred P-gp to cancerous cells (i.e., drug-sensitive MCF7). The presence of CD44 on the surface of EVs derived from resistant MCF7 cells and the absence of this surface marker on the EVs from resistant CEM cells was proposed as a possible explanation for this cell-specific transfer [115]. The pathophysiological relevance of transporter transfer to nontumoral cells still has to be elucidated. Furthermore, it should be noted that the set of nonmalignant cells used for this study was rather arbitrarily selected. It would be interesting, for instance, to investigate the effect of tumoral EVs on nontumoral cells in the tumor microenviroment, as the latter may also influence drug resistance [116] and, ultimately, cancer prognosis.

Transfer of drug resistance via MVs in human acute lymphoblastic leukemia was also reported in another study using a similar model. In this case, P-gp-containing MVs were isolated from drug-resistant CEM cells and added to a culture of drug-sensitive cells. As expected, an increase in P-gp protein expression in target cells was observed. Furthermore, target cells exhibited increased efflux of the P-gp substrate rhodamine 123 [117]. Transfer of functional P-gp was also observed in KB cells, originally classified as human oral epidermoid carcinoma [118]. Here, a vincristine-resistant clone was reported to express higher levels of P-gp. Furthermore, vincristine triggered a concentration-dependent increase in the P-gp content secreted in the EVs. In addition, using a coculture system in transwells, the authors demonstrated the transfer of functional P-gp from drug-resistant to drug-sensitive cells. Noteworthy, no P-gp mRNA was detected in target cells, even at high concentrations of EVs, thus supporting the transfer of P-gp only at the protein level from donor (resistant) to target (sensitive) cells. In addition, EVs from donor cells enhanced the resistance of target cells to doxorubicin, a well-known P-gp substrate, this process being prevented by incubation with the P-gp inhibitor verapamil. On the contrary, EVs did not modify the resistance to cisplatin. Mechanistically, the transfer of P-gp can be partially explained in terms of Rab8 upregulation in the drug-resistant cells upon exposure to vincristine, which leads to increased EV release. Furthermore, the EV uptake in the drug sensitive cells takes place via a clathrin-mediated endocytosis. Importantly, these findings were also confirmed in vivo using a mouse xenograft model [118]. These observations clearly demonstrate the transfer of a fully functional drug transporter via EVs, which also acquires a cellular localization in the target cell compatible with the efflux activity, and strongly suggest increased drug efflux as the mechanism underlying the stimulation of drug resistance by EVs.

Similar conclusions were obtained using osteosarcoma cells (MG-63). Doxorubicin-resistant cells with higher levels of P-gp mRNA produced EVs with higher content of this messenger. Treatment of sensitive MG-63 cells with these EVs resulted in an increase in P-gp mRNA expression in target cells. Concomitantly, an increase in resistance to doxorubicin was observed [119]. Noteworthy, in this study, several miRNAs from the drug-resistant cells were also transferred to the sensitive cells via EVs. This way, other cancer-related features such as invasion were modified by EVs. However, to date, there is no evidence relating these miRNAs to P-gp expression [119]. A similar study, also using drug-resistant and drug-sensitive MG-63 cells, reported the transfer of both P-gp and its coding mRNA with the concomitant enhancement of chemoresistance in target cells [120].

EV-mediated transfer of transporters has also been described in cancers of the digestive tract. In the gastric cancer cell lines HGC27 and KATOIII, paclitaxel-resistant clones with P-gp upregulation released EVs with higher P-gp content than sensitive clones. Furthermore, the addition of EVs from the resistant cells to the sensitive counterparts stimulated the resistance to paclitaxel, most likely due to enhanced drug efflux [121]. Also, EVs from drug-resistant HepG2 (i.e., hepatocellular carcinoma) cells increased the resistance of parental HepG2 cells to cisplatin. Mechanistically, EVs ameliorate the increase in ROS triggered by cisplatin. Furthermore, exposure to EVs increased the expression of P-gp in target cells. Similar findings were observed when EVs from resistant HepG2 cells were added to sensitive Huh7 and SMMC-7721 cells, also derived from hepatocellular carcinoma. Since the EVs from the resistant cells exhibited higher P-gp content than EVs from sensitive cells, an EV-mediated transfer of the transporter protein seems feasible [122]. However, whether higher P-gp expression after exposure to EVs is directly related to the enhanced resistance to cisplatin, or whether it belongs to a package of malignancy-related features transferred via EVs, as mentioned above [119], without directly contributing to the resistance to this particular drug has not been demonstrated. Similarly, proteomics analysis identified P-gp enrichment in EVs obtained from the pancreatic juice of patients with pancreatic adenocarcinoma as well as in EVs from pancreatic cancer cell lines capan-1 and MIA PaCa-2. The association between the expression levels of P-gp and therapy resistance was, however, not investigated [123]. Considering that EVs obtained from pancreatic juice are likely to derive from pancreatic tissue, this approach could be used to obtain tissue- and, eventually, tumor-specific information without interference from EVs of other tissues, as is the case for plasma-derived EVs. On the contrary, the sample collection would constitute a highly invasive procedure, the application of which may difficult in clinical practice.

In prostate cancer, EV-mediated transfer of P-gp and drug resistance has also been investigated. In fact, addition of EVs from docetaxel-resistant DU145RD and 22Rv1RD cells to parental clones (DU145 and 22Rv1) increased resistance to this cytostatic agent. The effect was negligible when the target DU145 and 22Rv1 cells were incubated with EVs obtained from the same drug-sensitive cells. In line with these findings, Western blot experiments demonstrated the upregulation of P-gp in both drug-resistant cell lines and their derived EVs. Noteworthy, incubation of DU145 and 22Rv1 cells with serum EVs from prostate cancer patients who did not respond to therapy with docetaxel also stimulated docetaxel resistance in target cells. On the contrary, incubation with serum EVs from responders enhanced sensitivity to docetaxel [124]. Since P-gp expression was neither determined in serum EVs nor in target cells after treatment with these EVs, the link between EV-mediated resistance and P-gp still needs to be confirmed.

While P-gp’s role in EV-mediated transfer of drug resistance has been extensively demonstrated, evidence involving other ABC transporters is rather limited. For instance, a proteomics analysis identified the overexpression of MRP1 and MRP4 in EVs from metastatic colorectal cancer cells (SW620) compared to primary tumor cells (SW480) [125]. Roles of MRP1 [126] and MRP4 [127] in colorectal cancer chemoresistance have been proposed. However, the participation of EVs in this process is still not confirmed. Transfer of MRP1 was also described in acute lymphoblastic leukemia cells (CCRF-CEM). Here, an MRP1-overexpressing clone (E_1000_) released MVs containing MRP1, which, once added to the parental CEM cells, resulted in MRP1 upregulation at the mRNA and protein level as well as in an increase in transporter activity [128]. In promyelocytic leukemia cells (HL60), treatment with EVs from a clone overexpressing MRP1 and resistant to daunorubicin (HL60/AR) transferred daunorubicin resistance to target cells. Experiments with the MK571 inhibitor also demonstrated an increase in MRP1 activity in EV-exposed HL60 cells. These findings also confirm the transfer of multidrug resistance in promyelocytic leukemia. Considering that the increase in MRP1 activity already takes place within a short incubation time (20 h), the authors proposed the EV-mediated direct transfer of transporter molecules from HL60 to HL/AR cells as the underlying molecular mechanism [129].

BCRP presence in circulating EVs was demonstrated in breast cancer patients. Moreover, plasma EVs from patients who did not respond to the neoadjuvant chemotherapy with anthracyclines and/or taxanes exhibited higher mRNA and protein levels in the transporter than EVs isolated from patients who responded to treatment [130]. Interestingly, the presence of BCRP in EVs has also been associated with decreased doxorubicin resistance in breast cancer. Contrary to the above-described mechanisms, an increase in cellular ceramide (e.g., by addition of exogenous ceramide, by inhibition of the sphingomyelin synthase, by incubation with the RXR agonist bexarotene combined with the FXR antagonist guggulsterone) increased BCRP in EVs released by MDA-MB-231 breast cancer cells, concomitant with a decrease in cellular BCRP protein levels and, most likely, an increase in the accumulation of its substrates. mRNA levels remained unchanged in the cells [131]. In addition to demonstrating the presence of BCRP in EVs released by cancer cells, this study clearly highlights the relevance of investigating the impact of EV signaling not only in target cells but also in donor cells.

Human acute myeloid leukemia cells (AML, U937 cells) exposed to plasma EVs from patients with newly diagnosed and recurrent AML exhibited an increase in idarubicin resistance concomitant with upregulation of P-gp and MRP1 mRNA expression. Importantly, incubation of U937 cells with plasma EVs from healthy volunteers did not lead to any changes in the transporter. Coincubation with actinomycin D prevented transporter induction. These findings clearly indicate transcriptional regulation upon the addition of vesicles and argue against the simple shuttling of transporter mRNA via the EVs [132]. The specific regulatory factors encapsulated in the EVs, which lead to an increase in transporter expression in target cells, have not been investigated. 

### 2.2. EV-Mediated Regulation of ABC Transporters

#### 2.2.1. MicroRNAs

##### Inhibition of Multidrug Resistance via EV-Carried MicroRNAs

Contrary to previous studies, where, mostly, the stimulation of drug-resistance by EVs was described, the opposite effect may also occur upon delivery of EV cargo. This is the case when the vesicles transport miRNAs targeting drug transporters, as demonstrated, for instance, in human acute lymphoblastic leukemia cells (CEM). The P-gp-overexpressing CEM clone (VLB_100_) releases EVs enriched in miR-326, which, upon addition to the MRP1-overexpressing clone E_1000_, led to MRP1 downregulation at the mRNA and protein levels. The effect was prevented by transfection of target cells with an miR-326 inhibitor, thus confirming the regulation of MRP1 by this miRNA [133].

Alternatively, miRNAs may regulate drug transporters in a more indirect way, for example, by targeting mRNAs codifying regulatory proteins. This was the case in gastric cancer cells. To establish this mechanism, SGC-7901 and MGC-803 drug-sensitive gastric cancer cells and SGC-7901/5FU cells, resistant to 5-fluorouracil (5-FU) and cisplatin, were used.

Treatment of SGC-791/5FU cells with SGC-7901 and MGC-803 EVs increased sensitivity to 5-FU and cisplatin. The prevention of the effect by treatment of donor cells with GW4869 clearly confirms the involvement of EVs in the transfer of the drug-sensitive phenotype. EVs from both drug-sensitive cells carried significantly higher levels of miR-107 than EVs from the drug-resistant clone. Moreover, miR-107 mimics reproduced the increase in chemosensitivity achieved by treatment of SGC-7901/5FU cells with EVs, further suggesting the sensitizing role of this miRNA. Treatment with SGC-7901 EVs also resulted in the downregulation of P-gp protein expression in target cells. Mechanistically, the authors proposed the downregulation of HMGA2 (high mobility group A2) by binding of miR-107 to its 3′UTR. This results in the inhibition of p-mTOR, which may be responsible for the downregulation of P-gp and increased drug sensitivity [134].

In MG-63 osteosarcoma cells, EVs from parental cells exposed to the flavonoid luteolin enhanced the sensitivity of the resistant clone MG-63/DOX to doxorubicin. Upregulation of miR-384 by luteolin has been proposed as one of the main factors leading to this outcome. In fact, miR-384 targets pleiotrophin (PTN), which may regulate P-gp expression via the β-catenin pathway. Using a mice xenograft model, increase in miR-384, downregulation of P-gp in the tumor cells, and reduced tumor growth due to the treatment with luteolin were also observed [135]. A similar process was proposed in MCF7 cells. Here, EVs from doxorubicin- and docetaxel-resistant clones (MCF7/ADR and MCF7/DOC, respectively) triggered drug resistance in drug-sensitive MCF7 cells. In this experimental setting, β-elemene, a vegetal compound used in traditional Chinese medicine, counteracted the development of resistance. The authors proposed a mechanism consisting in miR-34a upregulation and miR-452 downregulation in the EVs and downregulation of P-gp expression in drug-resistant cells. However, a complete molecular mechanism linking all these observations is still missing [136].

Nontumoral cells may also release EVs, which ultimately modulate drug resistance via miRNAs. Treatment of hepatocellular carcinoma HepG2 and Hep3B cells as well as primary tumor cells with EVs from human cerebral endothelial cells (hCECs) overexpressing miR-214 led to the sensitization to oxaliplatin and sorafenib. No sensitizing effect was observed when the cells were treated with EVs from hCECs without miR-214 overexpression, which clearly points to the regulatory role of this miRNA. Preincubation (i.e., priming) of HepG2 and Hep3B cells with EVs from hCECs overexpressing miR-214 also resulted in sensitization to both chemotherapeutic agents, although the magnitude of the effect was minimal compared to the simultaneous coincubation with EVs and drugs. Incubation with hCEC-miR-214 EVs also resulted in the downregulation of P-gp in HepG2 and Hep3B, probably contributing to the chemosensitizing effect [137]. A similar experimental approach was also used to sensitize glioblastoma multiforme (GBM) cells. GBM is characterized by high expression of miR-9, which is associated with malignancy and therapy resistance. In this context, Munoz et al. demonstrated the transfer of anti-miR-9 from mesenchymal stem cells (MSCs) to the U87 and T98G GBM cell lines in a contact-independent manner. The transfer inhibition by treatment of donor cells with manumycin A (i.e., inhibitor of EV release) confirms the participation of EVs. Direct treatment of GBM cells with anti-miR-9 led to P-gp downregulation [138]. Altogether, these findings clearly indicate the possibility of cell–cell communication between nontumoral and tumoral cells, via EVs, with an impact on drug resistance. However, while these delivery mechanisms may be experimentally exploited for the development of therapeutic strategies, both studies and the identified mechanisms relied on highly unphysiological models, which make difficult to elucidate whether they may influence drug resistance in vivo.

##### Stimulation of Multidrug Resistance via EV-Carried MicroRNAs

Delivery of miRNAs by EVs may ultimately result in transporter upregulation and development of a multidrug-resistant phenotype. In this regard, a study on breast cancer cell lines proposed miR-423-5p delivered by EVs as an indirect regulator of chemoresistance. In particular, EVs from cisplatin-resistant MDA-MB-231 cells (231/DDP) were added to the culture medium of drug-sensitive MDA-MB-231, MCF7, and SKBR3 breast cancer cells. As a result, an increase in the resistance to cisplatin concomitant with P-gp upregulation was observed. A miRNA analysis pointed to a significant enrichment in miR-423-5p in EVs from 231/DDP cells respect to EVs from the parental cells. In line with this, transfection of drug-sensitive cells with miR-423-5p mimics potentiated the effect of 231/DDP EVs in terms of P-gp upregulation and resistance to cisplatin while, on the contrary, transfection with a miR-423-5p inhibitor counteracted this effect. Altogether, the study clearly shows the stimulation of drug resistance by EVs. While the experimental evidence points to the role of P-gp and its regulation by miR-423-5p in EV-mediated resistance to cisplatin, further studies such as overexpression or silencing of miR-423-5p in donor 231/DDP cells would nicely provide final confirmation about this mechanism [139].

Participation of EV-delivered miR-9-5p, miR-195-5p, and miR-203a-3p in breast cancer multidrug resistance was also demonstrated. Treatment of MDA-MB-231 cells with docetaxel or doxorubicin led to an enrichment in these three miRNAs in EVs. Treatment of parental cells with these EVs induced sphere formation and led to significant downregulation of the transcription factor ONECUT2 (one cut homeobox 2) and the up-regulation of stemness related proteins (i.e., NOTCH1, SOX2, SOX9, NANOG, OCT4). The effects were prevented by simultaneous transfection of target cells with inhibitors of the abovementioned miRNAs. Knockdown experiments demonstrated the upregulation of P-gp, MRP1, and BCRP by decreased levels of ONECUT2. Also, a xenograft model in mice with wild type and Rab27A knockdown MDA-MB-231 cells (i.e., with reduced EV release) showed improved response to docetaxel in mice with deficient EV production. Altogether, the experimental evidence suggests the downregulation of ONECUT2 by miRNAs carried by EVs, which ultimately results in the induction of stemness-related proteins and drug transporters in target cells [140].

Prostate cancer cells also exhibited indirect transporter regulation by miRNAs. In this case, however, the resistance mechanism does not involve the shuttling of EV cargo between cells. Instead, treatment of DU145 cells with fludarabine resulted in changes in the release of different miRNAs to the extracellular space via EVs. While most of the analyzed miRNAs were enriched in EVs from treated cells, a decrease in the EV levels of miR-485-3p was observed. Concomitantly, an increase in the intracellular levels of miR-485-3p was also registered, suggesting a mechanism aimed at the conservation of this specific miRNA in the treated cells. In terms of drug resistance, miR-485-3p accumulation was associated with the downregulation of its target, the β subunit of the nuclear factor Y (NF-YB), which constitutes a negative regulator of P-gp transcription. In this way, a selective decrease in miR-485-3p in EVs and the consequent increased miR-485-3p levels in the cells lead to increased P-gp mRNA expression [141].

In ovarian cancer, EV-mediated transfer of miR-429 from drug-resistant SKOV3 cells to drug-sensitive A2780 led to acquisition of cisplatin resistance in target cells. The mechanism was confirmed using the EV release inhibitor GW4869 as well as a miRNA inhibitor. However, the participation of drug transporting proteins in the resistance mechanism was not investigated [142]. Similarly, EVs from drug-resistant hepatocellular carcinoma Bel7402 stimulated resistance to 5-FU, oxaliplatin, sorafenib, and gemcitabine in parental drug-sensitive Bel7402 cells. This effect was attributed to an enrichment in miR-32-5p in the EVs. Here, the participation of drug transporters also remains uninvestigated [143].

As mentioned in Inhibition of Multidrug Resistance via EV-Carried microRNAs Section, EVs from nontumoral cells may deliver miRNAs to cancer cells. Cases of increased drug resistance in target cells have hereby also been described. Higher levels of miR-301b-3p have been associated with gastric cancer drug resistance in vivo and in vitro. Treatment of SGC7901 gastric cancer cells resistant to cisplatin and vincristine with EVs from mesenchymal stem cells (MSCs) resulted in miR-301b-3p and P-gp upregulation. All the effects were counteracted by transfection of the donor MSC cells with anti-miR-301b-3p. Changes in the expression of drug transporters was attributed to the downregulation of TXNIP (thioredoxin-interacting protein) by EVs [144]. A similar effect, although with other molecular players, was observed in multiple myeloma cells. Here, MSC EVs delivered miR-155 to MPC-11 myeloma cells and increased P-gp, MRP1, and BCRP expression and resistance to bortezomib and dexamethasone. The molecular pathway linking the delivery of miR-155 to target cells and transporter regulation is not fully elucidated [145].

Also, cells from the tumor microenvironment release EVs, which may transfer miRNAs to tumor cells. This was the case in a model of coculture of epithelial ovarian cancer cells with M2 tumor-associated macrophages (M2 TAM). First, coculture of both cell types in a transwell system increased resistance to cisplatin in A2780 and A2780/DDP (i.e., already cisplatin-resistant) cells, suggesting the presence of a diffusible factor mediating cell–cell communication and being responsible for the effect. Treatment of A2780 and A2780/DDP cells with EVs isolated from M2 TAM led to diverse effects in terms of mRNA expression of drug transporters in target cells. Namely, P-gp was upregulated in A2780/DDP cells by treatment with EVs. P-gp upregulation by EVs was also observed in A2780 cells, although only in the presence of cisplatin. MRP1 and BCRP were slightly upregulated by M2 EVs in A2780 cells, while no changes were observed in A2780/DDP cells. miR-221-3p, which was enriched in M2 EVs with respect to monocytes from peripheral blood, was proposed as an important regulator of the drug resistance in this model [146].

Conversely, delivery of miRNAs from tumor cells to M2 TAMs was also demonstrated in ovarian cancer. In particular, EVs from SKOV3-ip1 cells increased miR-1246 levels in M2 and decreased the expression of caveolin-1 mRNA. Prevention of the effect by treatment of SKOV3-ip1 cells with GW4869 strongly indicates the participation of EVs. Although the link was not directly investigated, the inverse association between caveolin-1 and P-gp expression strongly suggests the regulation of P-gp levels by EV-carried miR-1246 in M2 macrophages [147]. Another study demonstrated the delivery of miR-1246 from highly metastatic melanoma cells (A375SM) to tumor endothelial cells. Increased resistance to 5-FU was also observed. However, the expression of drug transporters was not investigated in this case [148].

#### 2.2.2. Other Noncoding RNAs

Besides miRNA, EVs may also carry other noncoding RNAs such as circular RNAs (circRNAs), which can function as competing endogenous RNAs and inhibit the effect of miRNAs. This was the case in a study in non-small cell lung cancer (NSCLC). Here, EVs from A549 and H1299 cells were reported to deliver circ_PIP5K1A (hsa_circ_0014130) to the same A549 and H1299 cells and stimulate resistance to cisplatin. Once in the target cells, circ_PIP5K1A binds to and impairs the regulatory function of miR-101. Since MRP1 is a target mRNA of miR-101, upregulation of this drug transporter by EV-carried circ_PIP5K1A can be expected and is likely to account for the resistance to cisplatin. From the clinical point of view, serum EVs from NSCLC patients exhibited higher levels of circ_PIP5K1A than EVs from healthy volunteers [149]. A similar study also identified circ_0076305, which was enriched in EVs from NSCLC cells, as an endogenous competitor of miR-186-5p and, therefore, a positive regulator of MRP1 expression and resistance to cisplatin [150].

In osteosarcoma, EVs from cisplatin-resistant MG-63/CDDP cells exhibited a higher content of hsa_circ_103801 than parental MG-63 cells. In addition, MG-63/CDDP EVs triggered an increase in hsa_circ_103801, cisplatin resistance, P-gp, and MRP1 expression in drug-sensitive osteosarcoma cells (MG-63 and U2OS). This effect was strongly enhanced by overexpression of hsa_circ_103801 in donor cells [151]. In gastric cancer, oxaliplatin-resistant cells (HGC27/OXA) expressed higher levels of the hsa_circ_0091741 than drug-sensitive HGC27 cells. Treatment of the latter with HGC27/OXA EVs stimulated hsa_circ_0091741 expression and led to P-gp, MRP1, and LRP1 (lung resistance protein 1) upregulation at the mRNA level. The effect was associated with a decrease in miR-330-3p expression, probably mediating the effects of the circRNA on the different target genes [152].

Long noncoding RNAs (lncRNA) also belong to EV cargo and modulate drug resistance. In this regard, EVs from cutaneous squamous cell carcinoma (CSCC, HSC-5) exhibited higher levels of the lncRNA PICSAR (p38-inhibited cutaneous squamous cell carcinoma-associated lincRNA), while these levels were further increased in the case of cisplatin-resistant HSC-5 cells. In line with this, serum EVs from CSCC patients showed higher PICSAR levels than serum EVs from healthy volunteers. Studies in CSCC cells showed the inhibition of miR-485-5p by PICSAR, which in turn results in the upregulation of P-gp and MRP1 protein levels [153]. Similarly, esophageal carcinoma cells Eca109 exposed to increasing concentrations of doxorubicin produced EVs with increasing content of the LncRNA VLDLR. Treatment of Eca109 cells with EVs released by these pretreated cells resulted in the development of doxorubicin resistance, thus indicating the transfer of the drug-resistant phenotype from the donor to the target cells [154].

As demonstrated for miRNAs, circRNAs can, instead, induce drug sensitivity in target cells. In colorectal cancer cells, treatment of oxaliplatin-resistant clones SW480/OxR and HCT116/OxR with EVs derived from normal colon cells overexpressing circ_FBXW7 led to increased expression in the circRNA in target cells. Also, increased sensitivity to oxaliplatin and increased intracellular platinum accumulation were observed. miR-18b-5p and MRP1 protein expression were also downregulated in target cells. All the effects were counteracted by transfection of the target cells with miR-18b-5p mimics. In vivo, using a mice xenograft model based on HCF116/OxR cells, intratumoral injection of EVs carrying circ_FBXW7 led to downregulation of MRP1 protein levels and reduced tumor growth in the presence of oxaliplatin. In this way, an inhibitory effect of circ_FBXW7 on miR-18b-5p appears as a feasible mechanism leading to decreased drug resistance. The steps linking miR-18b-5p and MRP1 regulation have not been elucidated yet [155].

#### 2.2.3. Regulatory Proteins

EVs can also carry regulatory proteins from a donor (usually drug-resistant) cell to a target cell, where they upregulate drug transporters and stimulate multidrug resistance. The EV components leading to the modulation of drug transporters may be known or unknown. For instance, the treatment of gastric cancer cells with EVs derived from MSCs resulted in P-gp, MRP1, and LRP upregulation and the stimulation of resistance to 5-FU. Both effects were blocked by treatment with the MEK/ERK inhibitor U0126 and with the Raf1 inhibitor vemurafenib. Furthermore, treatment of EVs with proteinase K but not with RNAse A prevented the increase in transporter expression and the resulting chemoresistance. EVs from MSCs with P-gp knockdown still resulted in transporter upregulation. These findings point to the induction of transporter expression in gastric cancer cells via the protein cargo of MSC EVs. Although a direct shuttle of P-gp from MSCs to gastric cancer cells can be ruled out based on the knockdown results, the specific proteins transported by the EVs and responsible for the effect in the target cells have not been elucidated [156].

On the contrary, several studies have succeeded in identifying components within the EV cargo responsible for transporter regulation. This was the case, for example, in MCF7 breast cancer cells. In particular, doxorubicin-resistant cells (MCF7/ADM) released EVs with higher levels of the protein UCH-L1 (ubiquitin carboxy-terminal hydrolase L1), which regulates P-gp expression via activation of the MAPK/ERK pathway. Once added to drug-sensitive MCF7 cells, MCF7/ADM EVs led to UCH-L1 upregulation, increased p-ERK phosphorylation, P-gp upregulation, and resistance to doxorubicin. Since the effects were prevented by preincubation of the EVs with the UCH-L1 inhibitor LDN57444, the development of drug resistance is most likely to occur via P-gp induction in target cells via EV-carried UCH-L1 instead of by a direct transfer of P-gp from the donor to the target cells [157].

Another study using MCF7/ADM cells demonstrated a key role of transient receptor potential channel 5 (TrpC5) in EV-mediated multidrug resistance. Here, MCF7/ADM cells released EVs with higher TrpC5 and P-gp mRNA levels. Addition of MCF7/ADM EVs to the culture medium of parental MCF7 cells resulted in TrpC5 overexpression, P-gp upregulation, and resistance to doxorubicin. Interestingly, the increase in P-gp expression in the target cells was totally prevented by incubation of MCF7/ADM EVs with an antibody against TrpC5, thus pointing to a regulatory role of the latter on P-gp expression, rather than a direct transfer of the drug transporter. Furthermore, TrpC5 was detected in circulating EVs obtained from mice bearing MCF7/ADM tumors as well as from breast cancer patients already exposed to chemotherapy. TrpC5 was not detected in circulating vesicles from therapy-naïve patients. Thus, these findings indicate the transfer of TrpC5 as an important mechanism stimulating multidrug resistance in target cells [158]. The same mechanism was demonstrated after addition of MCF7/ADM microvesicles to human microvessel endothelial cells (HMECs). Here, again, treatment of target cells with MCF7/ADM MVs led to TrpC5 and P-gp upregulation. As in the previous model, P-gp upregulation was prevented by inhibition of TrpC5 in the MVs, which suggests the major regulatory role of this protein. Mechanistically, TrpC5 delivered via MVs is proposed to increase cell permeability to Ca^2+^, which results in the activation and binding of transcription factor NFATc3 (nuclear factor of activated T cells) to the P-gp promoter, which leads, ultimately, to the induction of P-gp transcription [159].

Nrf2 is a transcription factor already demonstrated to regulate P-gp expression [160]. Human colorectal cancer cells resistant to oxaliplatin LS174T/R express higher levels of Nrf2 than their drug-sensitive counterpart (LS174T). Furthermore, experiments with the Nrf2 inhibitor brusatol demonstrated the association between Nrf2 and drug resistance. Moreover, EVs released by LS174T/R cells exhibited higher Nrf2 levels than EVs released by drug-sensitive cells. Incubation of LS174T cells with LS174T/R EVs resulted in Nrf2 overexpression concomitant with P-gp upregulation at the protein level, increased P-gp activity (determined based on the rhodamine 123 efflux), and increased resistance to oxaliplatin [161]. Considering the well-known mechanism of P-gp regulation by Nrf2 [160], delivery of this nuclear factor by LS174T/R to LS1774T cells by EVs is likely to be the main factor leading to multidrug resistance in this model. This may result in P-gp induction at the transcriptional level and increase in the P-gp protein levels with a concomitant stimulation of drug resistance, as observed by the authors. The link between upregulated P-gp and enhanced efflux of oxaliplatin was, however, not investigated [161]. In another study, using SW480 and SW620 colon adenocarcinoma cells, resistance to oxaliplatin was associated with higher levels of the regulatory protein DNAJB8, which positively regulates P-gp expression via the interaction with TP53. Drug-resistant cells produced EVs with higher levels of DNAJB8 than their sensitive counterparts. Moreover, incubation of sensitive cells with EVs from resistant clones triggered resistance to oxaliplatin in target cells. The transfer of DNAJB8 via EVs was confirmed in experiments using proteinase K and triton in order to disrupt the EVs as well as using EVs from donor cells with DNAJB8 knockdown [162]. In line with these findings, another study associated DNAJB8 with enhanced resistance to docetaxel, also a P-gp substrate, in renal clear cell carcinoma [163]. Further studies should be performed to elucidate whether DNAJB8 transfer via EVs indeed results in an increase in P-gp-mediated drug efflux in target cells. If this is the case, EV-mediated transfer of DNAJB8 may be related to resistance toward several other chemotherapeutic agents transported by P-gp.

In esophageal squamous cell carcinoma, regulation of drug resistance via EVs was observed using paclitaxel-sensitive and paclitaxel-resistant cell lines (EC-9706 and EC-9706R, respectively). The authors identified a higher content of PD-L1 (programmed death ligand 1) in EVs from EC-9706R cells. EC-9706R EVs also exhibited higher P-gp levels than EVs from drug-sensitive cells. Treatment of EC-9706 cells with EC-9706R EVs increased resistance of target cells to paclitaxel. Furthermore, incubation of EC-9706R cells with the EV-release inhibitor GW4869 impaired drug resistance. In line with this, treatment of a mice xenograft model established with EC-9706 cells with EC-9706R EVs resulted in the upregulation of PD-L1, STAT3, and miR-21. The authors proposed a model in which PD-L1 delivered by EVs to sensitive cells results in P-gp upregulation via the p-STAT3/miR-21 pathway [164]. Although the direct association between miR-21 expression and P-gp activity and function was clearly demonstrated in other models [165,166] and is likely to be, at least in part, responsible for resistance to paclitaxel, a direct association was not demonstrated in this study [164].

In nasopharyngeal carcinoma cells (CNE1), a paclitaxel-resistant clone overexpressed P-gp as well as the regulatory protein DDX53 (DEAD-Box helicase 53). Interestingly, coculture of resistant and sensitive cells separated through a porous membrane resulted in the upregulation of both DDX53 and P-gp in drug-sensitive cells. Concomitantly, an increase in resistance to paclitaxel was observed. Further experiments confirmed that drug-resistant cells produced EVs with higher expression of DDX53 and P-gp. In addition, coculture in the presence of GW4869 (i.e., inhibitor of EV release) prevented DDX53 and P-gp upregulation in the sensitive cells. Resistance to paclitaxel was also prevented by GW4869. Altogether, these findings clearly demonstrate the EV-mediated transfer of a drug-resistant phenotype via the shuttle of a regulatory protein such as DDX53. Interestingly, other transporters such as MRP1 and MRP2 were not upregulated in drug-resistant cells, thus pointing to a solely P-gp-dependent resistance mechanism [167]. Direct transfer of P-gp to drug-sensitive cells cannot be totally ruled out.

In lung adenocarcinoma, gefitinib-resistant cells (PC9/GR and H1975) exhibited higher levels of the N6-methyladenosine (m^6^A) eraser FTO (fat mass and obesity-associated protein). In line with this, FTO was overexpressed in serum EVs from gefitinib-resistant lung cancer patients compared to serum EVs from gefitinib-sensitive patients. Also, treatment of PC9 gefitinib-sensitive cells with PC9/GR and H1975 EVs increased resistance to the drug, concomitant with a decrease in m^6^A content in target cells. Since the effect was prevented by FTO knockdown in donor cells, a major role of FTO carried by EVs can be proposed. At the transporter level, the levels of BCRP and ABCC10 (i.e., multidrug resistance-associated protein 7, MRP7) in target cells were directly associated with FTO levels in EVs. Changes in m^6^A levels in the *ABCC10* gene were also demonstrated experimentally; m^6^A levels in the *ABCG2* gene (codifying BCRP) were not investigated [168]. Altogether, FTO-carrying EVs released from drug-resistant cells appear to result in FTO upregulation in drug-sensitive cells. This may lead to changes in the methylation status of transporter-codifying genes, a concomitant increase in transporter expression, and, ultimately, enhanced drug resistance. Table 1 and Table 2 summarize the cases of stimulation and inhibition of drug resistance through EV-mediated cell–cell communication, respectively.

### 2.3. Drug Sequestration in EVs

Besides the regulation of drug transporters, EVs may play a role in drug resistance by sequestering chemotherapeutic agents. Kavanagh et al. demonstrated the sequestration of Flutax-2 (a fluorescent analogue of paclitaxel) in EVs released by senescent triple-negative breast cancer cells. Furthermore, senescent cells, due to prolonged exposure to paclitaxel, released a higher number of EVs than the nonsenescent control cells. Concomitantly, an increase in P-gp expression in senescent cells but, on the contrary, a decrease in P-gp levels in the derived EVs were observed. The authors proposed a model wherein senescent cells use EVs as means of excretion for cytotoxic drugs and proapoptotic proteins. On the contrary, the amount of P-gp lost by the cell via EVs is lower in senescent cells, thus guaranteeing higher transporter expression in the cell in order to prevent intracellular drug accumulation [169]. Another study on breast cancer MCF7 cells demonstrated the sequestration of doxorubicin in EVs released by the doxorubicin-resistant clone MCF7/ADR, while no doxorubicin was detected in the EVs from sensitive MCF7 cells. MCF7/ADR EVs exhibited higher P-gp expression than the vesicles derived from drug-sensitive cells and simultaneously mediated the transfer of the transporter to drug-sensitive cells. However, it is not clear whether P-gp itself is responsible for the sequestration of doxorubicin inside EVs [112]. In another study, MVs obtained from doxorubicin-resistant MCF7 cells exhibited sequestration of doxorubicin in MVs due to active pumping by P-gp located on the MV membrane in an inside-out configuration. Nevertheless, MVs from parental MCF7 cells, which do not express detectable levels of P-gp, exhibited significantly higher doxorubicin and daunorubicin sequestration than vesicles from resistant cells. The authors postulated the larger vesicle size, as well as the higher concentration of phospholipids and nucleic acids in the parental cell vesicles, as a potential factor stimulating passive drug accumulation in these MVs [170]. In summary, although drug sequestration in breast cancer EVs is well demonstrated, the relevance of P-gp in this process and to what extent these drug sequestration mechanisms contribute to overall drug resistance are still unclear.

Another study identified the formation of large EV-like structures in the cell–cell attachment regions of MCF7/MR cells (i.e., resistant to mitoxantrone) by using immunohistochemistry and electron microscopy. Furthermore, MCF7/MR cells exhibited a larger number of these structures per number of cell than in the parental MCF7 cells. BCRP expression was detected on the surface of EV-like structures as well as mitoxantrone accumulation inside the vesicles, which was prevented by the BCRP inhibitors Ko143 and fumitremorgin C [171]. Unlike previous studies [112,169,170], in this case, the participation of a drug transporter (i.e., BCRP) in drug sequestration is strongly supported by experimental evidence. Furthermore, the same group also demonstrated the role of Akt phosphorylation stimulating the localization of BCRP on the surface of the EV-like structures. In fact, blocking of Akt signaling with LY294002 decreased the concentration of BCRP on the EV surface and increased the concentration of BCRP in the cytosol of MCF7/MR cells. Simultaneously, an increase in sensitivity to mitoxantrone and topotecan by LY294002 was observed [172]. Altogether, this novel mechanism of resistance proves the clear role of an ABC transporter in drug sequestration in EVs. Further studies should be performed to investigate whether the same process also takes place in more complex systems such as organoids or tumors and whether it can contribute to drug resistance in a clinical setting.

Hepatocellular carcinoma cells resistant to 5-FU (Bel7402/5Fu) exhibited accumulation of this drug in EVs. No 5-FU accumulation was observed in EVs derived from parental drug-sensitive Bel7402 cells. Moreover, treatment with 5-FU increased EV release and up-regulated the small GTPase Rab27B, which appears to play a major role in drug resistance, as demonstrated in overexpression and knockdown experiments. In the same study, Rab27B knockdown resulted in the upregulation of P-gp. Overall, the role of Rab27B in the resistance to 5-FU, which was also demonstrated in a xenograft model in mice, is strongly supported by experimental evidence. However, the net effect of 5-FU sequestration in EVs to the overall resistance and, moreover, the role of P-gp in this process are still unclear [173].

In another study using human ovarian carcinoma cells (OV2008), a drug-resistant subclone produced EVs with a higher content of cisplatin than drug-sensitive cells. Furthermore, EVs from the resistant clone carried higher levels of MRP2, a cisplatin transporter. However, the significance of this process in the resistance mechanism is also unclear. For instance, the amount of cisplatin extruded via EVs is very low compared with the total intracellular content of cisplatin. Furthermore, it is not clear whether MRP2 may play a role in the transport of cisplatin to EVs [174]. In addition, it still has to be elucidated whether the presence of higher levels of a cisplatin transporter, such as MRP2 in EVs from resistant cells, may contribute to the dissemination of cisplatin resistance, as demonstrated for other transporters and drugs (see Section 2.1).

In summary, although drug sequestration in EVs is well established, the participation of ABC transporters in this process, with the exception of BCRP in mitoxantrone-resistant cells, is not yet confirmed [171,172]. In addition, the body of evidence supporting the clinical relevance of this mechanism is not as strong as for other EV-mediated drug resistance mechanisms.

## 3. EVs as Biomarkers of Multidrug Resistance

Due to their high abundance and easy accessibility in biological fluids such as blood and urine, EVs bear a high potential as source of biomarkers. In this regard, it should be noted that the lack of standardized EV isolation and analysis methods has, so far, delayed their application as liquid biopsy. Nevertheless, several studies, usually with small patient cohorts, strongly suggest an association between specific components of EV cargo and drug resistance in a clinical setting.

For instance, the proportion of BCRP^+^ MVs in serum from breast cancer patients with axillary lymph node metastasis was higher than in serum from patients without positive lymph nodes or patients with benign tumors. Analysis of MVs among patients with different tumor sizes identified a higher proportion of BCRP^+^ MVs in the serum of patients with small tumors (T1) compared to patients with benign disease. All samples were obtained before administration of chemotherapy [175]. In addition, other authors demonstrated higher levels of BCRP mRNA in serum EVs from patients unresponsive to chemotherapy (anthracycline and/or taxane) with respect to therapy-naïve patients [130]. Since several drugs used in breast cancer chemotherapy are BCRP substrates, these findings point to the role of EVs in the prediction of breast cancer response to chemotherapy. In fact, the correlation between the cargo of EVs and BCRP function in the tissue of origin was already demonstrated in a study with healthy volunteers. Here, a positive correlation between the content of miR-328 (inversely associated with BCRP expression [176]) in intestinal EVs isolated from serum and the area under the curve of orally administered sulfasalazine (BCRP substrate) was identified [177]. Altogether, these data highlight the presence of BCRP in EVs isolated from blood and indicate the association between the cargo (either in terms of the transporter itself or transporter-regulating factors) of whole or partial populations of plasma EVs and the transporter function in the tissues of origin.

Also, P-gp has been detected in serum EVs. In fact, Kato et al. observed significantly higher P-gp protein levels in EVs from a cohort of docetaxel-resistant prostate cancer patients compared to EVs from patients not exposed to chemotherapy [178]. In the same line, higher protein levels of P-gp were observed in serum EVs from patients with docetaxel-resistance prostate cancer respect to patients with docetaxel-sensitive disease [179]. Although the number of patients in both studies was relatively small (between three and six), these observations support the potential of analyzing P-gp in EVs from cancer patients to predict the treatment outcome. Noteworthy, the analysis may also be extended to P-gp-regulatory proteins transported by EVs. This was the case for UCH-L1 in EVs, for which significantly higher levels of the protein were detected in plasma EVs from breast cancer patients, who did not respond to a chemotherapy scheme with anthracyclines and/or taxane [157]. Higher levels of miR-1246 related to P-gp expression have been described in plasma from melanoma patients with respect to healthy volunteers [148]. However, correlation with therapy response still has to be determined. Instead, analysis of DNAJB8, involved in P-gp regulation, in serum EVs from colon cancer patients who did not respond to therapy with oxaliplatin showed higher levels of this protein compared with responder patients [162].

In a rather indirect assay, Corcoran et al. investigated the potential of serum EVs from prostate cancer patients to stimulate docetaxel resistance in prostate cancer cells in vitro. Interestingly, EVs from patients with higher PSA levels (i.e., poorer response to chemotherapy) elicited a stronger induction of drug resistance than EVs from patients with lower PSA levels (i.e., better response to chemotherapy). Although the indirect nature of the assay significantly reduces its potential application in the routine analysis, it strongly supports the predictive role of analyzing prostate cancer EVs and their cargo as indicators of the response to chemotherapy [124].

The stability of EV cargo during sample isolation and processing constitutes one of the challenges to be addressed while analyzing EV components in biological samples. Here, circRNAs exhibit a significant advantage with respect to other more unstable RNAs [180]. In this regard, the previously mentioned study on circ_103801 in osteosarcoma [151], where an association between this circRNA and drug resistance in vitro was established, also identified a correlation between the content of circ_103801 in serum EVs and the tumor size and stage. Also, a poorer overall survival was registered in patients with higher content of circ_103801 in serum EVs. The direct association, however, with drug transporters and chemotherapy response, as determined in vitro, still has to be confirmed [151]. In the same line, the lncRNA PICSAR, which was associated with multidrug resistance in vitro [153], was enriched in serum EVs from cutaneous squamous cell carcinoma patients with respect to serum from healthy volunteers [153]. Additionally, higher levels of circ-PIP5K1A, related to MRP1 expression, were observed in serum EVs from NSCLC patients with respect to EVs from healthy volunteers [149]. In both cases, again, correlation between levels of circRNA in serum EVs and response to chemotherapy still has to be determined.

Overall, although the potential of EVs as an easily accessible source of biomarkers is undeniable, so far, evidence obtained in the frame of clinical trials has not led to the development of clinically validated markers. Future research should focus on confirming the prognostic or diagnostic value of the biomarkers identified in this section using larger patient cohorts. Additionally, in some cases, the correlation between the levels of a component of the EV cargo and the therapy response still has to be established. Furthermore, standardization in terms of sample collection and processing and EV isolation and analysis still requires significant development.

## 4. Future Perspectives

During the last years, participation of EVs in the stimulation and inhibition of drug resistance has been demonstrated in several types and stages of cancer. Despite the large number of publications addressing the relevance of EVs and particular components of their cargo in the transfer and acquisition of the resistant phenotype, the number of studies clearly elucidating all the cellular and molecular players, the specific components of the EV cargo mediating the effects, their mechanism of action in the target cell, and the final impact on drug resistance is rather limited. Additionally, most studies have delivered evidence mostly obtained in vitro and confirmed only a few key findings in vivo. In most of the cases, confirmation of the effect of EVs in vivo is still missing. Here, the use of xenograft models based on cell knockout for specific components of EV cargo can provide further insight into the underlying mechanisms. In this regard, bilateral tumor models [181,182] could be a useful setting for investigating EV-mediated effects between drug-resistant and drug-sensitive cells within the same animal. Alternatively, the use of organ-on-a-chip and tumor-on-a-chip platforms may also provide clarifying data [183,184].

In any case, the benefit of using the previously mentioned models cannot be fully achieved if the components of the EV cargo influencing drug resistance are not totally identified. Here, techniques such as proteomics [125] and RNA-seq [185,186] from EVs from drug-resistant and drug-sensitive cells, or in the presence and absence of chemotherapy, may deliver useful information. Further evidence can also be obtained by applying proteomics and RNA-seq approaches to circulating EVs. Here, separation of tumor-specific circulating EVs [187] can reduce the interference from all the other populations of circulating vesicles.

Once clear EV-mediated communication pathways are identified, it should be determined whether they represent druggable targets. The participation of EVs in several physiological processes throughout the organism constitutes a challenge to overcome toward developing safe therapeutic strategies targeting EV-mediated signaling. In this regard, compounds acting on EV biogenesis, such as GW4869, constitute useful experimental tools but would have reduced potential to be used in the clinical practice. Here, it would be important to elucidate, first, whether EV-mediated transfer of drug resistance is a cell-specific process and, if this is the case, to identify the cell-specific factors governing this transfer, as partially performed, for example, for MCF7 and CEM EVs [115]. Additionally, considering that EVs can also stimulate chemosensitivity, as described in the previous sections, it is important that potential therapeutic strategies are aimed at specific interactions and components of the EV cargo leading to resistance.

Finally, the use of EVs as source of biomarkers is another field with enormous development potential. In addition to advances in the field of pharmacogenomics, nowadays, the estimation of drug transporter expression and the drug excretion capacity of a normal or tumoral tissue, as well as their changes, would require the application of highly invasive procedures (e.g., biopsies), strongly limiting any clinical application. Here, EVs constitute promising sources of biomarkers. Their presence in blood and urine would provide simple and non- or minimally invasive access, even if highly repetitive sampling is required, for example, to assess the impact of a certain therapy on the transporter levels. Here, one of the challenges is determining to what extent the levels of ABC transporters in the vesicles constitute a valid surrogate of the levels of transporters in the tissues. Moreover, it should be determined whether EVs could be used as dynamic surrogates, which also reflect changes in the transporter levels due to, for example, exposure to inducers, as is the case for several therapeutic agents. Finally, development and establishment of EVs as biomarkers still requires substantial research and improvement toward the standardization of isolation and analytical procedures [188].

## 5. Conclusions

Recently, a significant development in the field of EVs related to the pathogenesis of malignant diseases, including their role in the stimulation and spreading of MDR, has been achieved. In terms of the bioactive components of EV cargo, regulation of MDR can mostly be attributed to the transfer of proteins and RNAs from donor to target cells. Nevertheless, an unequivocal association between a particular protein or RNA and a phenotypical change in terms to chemoresistance is frequently missing. Future research also needs to investigate the specificity of the effects (e.g., whether they may also take place under physiological conditions in healthy tissues). In addition, recently identified mechanisms need to be confirmed in more complex models. If these aspects are successfully addressed, transfer of drug resistance via EV-mediated cell–cell communication will definitely become a druggable mechanism toward preventing or counteracting therapy failure due to transporter overexpression. 

## Figures and Tables

**Table 1:** Stimulation of drug resistance through EV-mediated cell–cell communication. Described are cases where EVs released by donor cells led to the stimulation of multidrug resistance upon addition to target cells. The relevant EV cargo, at least partially responsible for the effects, if known, is also indicated.

Donor Cell	Target Cell	Relevant EV Cargo	Effect on Target Cell	Reference
Drug-resistant MCF7 cells	Drug-sensitive MCF7 cells	P-gp transporter	↑ P-gp protein expression and activity ↑ Doxorubicin resistance.	[111]
Drug-resistant MCF7 cells	Drug-sensitive MCF7 cells	P-gp transporter	↑ P-gp and MRP1 mRNA expression ↑ Doxorubicin resistance	[112]
Docetaxel-resistant MCF7 cells	Drug-sensitive MCF7 cells	P-gp transporter	↑ P-gp protein expression ↑ Resistance to docetaxel	[113]
Drug-resistant MCF7	Drug-sensitive MCF7	P-gp mRNA	↑ P-gp mRNA	[114]
Drug-resistant CEM cells	Drug-sensitive CEM cells	P-gp mRNA, miR-326	↑ P-gp mRNA ↓ MRP1 mRNA	[114]
Drug-resistant CEM cells	Cancerous (drug-sensitive CEM cells) and noncancerous cells (human mammary basal epithelial cells, human osteoblasts, human urothelial cells)	P-gp and MRP1 transporters	↑ P-gp and MRP1 protein expression	[115]
Drug-resistant MCF7 cells	Drug-sensitive MCF7 cells	P-gp transporter, CD44	↑ P-gp protein expression	
Drug-resistant CEM cells	Drug-sensitive CEM cells	P-gp transporter	↑ P-gp protein expression ↑ P-gp activity	[117]
Vincristine-resistant KB cells	Drug-sensitive KB cells	P-gp transporter	↑ P-gp protein expression ↑ P-gp activity ↑ Resistance to doxorubicin	[118]
Doxorubicin-resistant MG-63 cells	Drug-sensitive MG-63	P-gp transporter and mRNA	↑ P-gp mRNA expression ↑ Resistance to doxorubicin	[119,120]
Paclitaxel-resistant HGC27 and KATOIII cells lines	Drug-sensitive HGC27 and KATOIII cell lines	P-gp transporter	↑ Resistance to paclitaxel	[121]
Drug-resistant HepG2 cells	Drug-sensitive HepG2 cells, Huh7, SMMC-7721 cells	P-gp transporter	↑ P-gp protein expression ↑ Resistance to cisplatin	[122]
Docetaxel-resistant DU145 and 22Rv1 cells	Drug-sensitive DU145 and 22Rv1 cells	P-gp transporter	↑ Resistance to docetaxel	[124]
Serum EVs from prostate cancer patients who did not respond to chemotherapy	Drug-sensitive DU145 and 22Rv1 cells	?	↑ Resistance to docetaxel	
MRP1-overexpressing CCRF-CEM cells	CCRF-CEM cells	MRP1 transporter	↑ MRP1 mRNA expression ↑ MRP1 protein expression ↑ MRP1 activity	[128]
MRP1-overexpressing and daunorubicin-resistant HL60/AR cells	Drug-sensitive HL60 cells	MRP1 transporter	↑ Resistance to daunorubicin ↑ MRP1 activity	[129]
Plasma EVs from patients with newly diagnosed and recurrent AML	U937 cells	?	↑ Resistance to idarubicin ↑ P-gp and MRP1 mRNA expression	[132]
Cisplatin-resistant MDA-MB-231 cells	MDA-MB-231, MCF7, SKBR3 cells	miR-423-5p	↑ P-gp expression ↑ Resistance to cisplatin	[139]
MDA-MB-231 cells treated with docetaxel or doxorubicin	MDA-MB-231 cells	miR-9-5p, miR-195-5p, miR-203a-3p	↑ P-gp, MRP1, and BCRP expression	[140]
Drug-resistant SKOV3 cells	Drug-sensitive A2780 cells	miR-429	↑ Resistance to cisplatin	[142]
Drug-resistant Bel7402 cells	Drug-sensitive Bel7402 cells	miR-32-5p	↑ Resistance to 5-FU, oxaliplatin, sorafenib, and gemcitabine	[143]
MSC	Cisplatin- and vincristine-resistant SGC7901 cells	miR-301b-3p	↑ P-gp expression	[144]
MSC	MPC-11 cells	miR-155	↑ P-gp, MRP1, and BCRP expression ↑ Resistance to bortezomib and dexamethasone	[145]
EVs from M2 tumor-associated macrophages (M2)	A2780 cells	miR-221-3p	↑ P-gp expression (in the presence of cisplatin) ↑ MRP1 and BCRP expression	[146]
	Cisplatin-resistant A2780 cells (A2780/DDP)		↑ P-gp expression	
SKOV3-ip1 cells	M2 macrophages	miR-1246	↑ Caveolin-1 mRNA expression	[147]
A375SM cells	Tumor endothelial cells	miR-1246	↑ Resistance to 5-FU	[148]
A549 and H1299 cells	A549 and H1299 cells	circ_PIP5K1A (hsa_circ_0014130)	↑ Resistance to cisplatin	[149]
A549 and H1299 cells	A549 and H1299 cells	circ_0076305	↑ MRP1 expression ↑ Resistance to cisplatin	[150]
Cisplatin-resistant MG-63/CDDP cells	Drug-sensitive MG-63 and U2OS cells	hsa_circ_103801	↑ Resistance to cisplatin ↑ P-gp and MRP1 expression	[151]
Oxaliplatin-resistant HGC27 cells	Drug-sensitive HGC27 cells	hsa_circ_0091741	↑ P-gp, MRP1, and LRP1 expression	[152]
Cisplatin-resistant HSC-5 cells	Drug-sensitive HSC-5 cells	lnc PICSAR	↑ P-gp and MRP1 expression	[153]
Eca109 cells (treated with doxorubicin)	Eca109 cells (untreated)	lncRNA VLDLR	↑ Resistance to doxorubicin	[154]
MSC	Gastric cancer cells	?	↑ P-gp, MRP1, and LRP expression ↑ Resistance to 5-FU	[156]
Doxorubicin-resistant MCF7 cells	Drug-sensitive MCF7 cells	UCH-L1	↑ P-gp expression ↑ Resistance to doxorubicin	[157]
Doxorubicin-resistant MCF7 cells	Drug-sensitive MCF7 cells	TrpC5 and P-gp mRNA	↑ P-gp expression ↑ Resistance to doxorubicin	[158]
Doxorubicin-resistant MCF7 cells	HMECs	TrpC5	↑ P-gp expression	[159]
Oxaliplatin-resistant LS174T cells	Drug-sensitive LS174T cells	Nrf2	↑ P-gp expression ↑ P-gp activity ↑ Resistance to oxaliplatin	[161]
Oxaliplatin-resistant SW480 and SW620 cells	Drug-sensitive SW480 and SW620 cells	DNAJB8	↑ P-gp expression ↑ Resistance to oxaliplatin	[162]
Paclitaxel-resistant EC-9706 cells	Drug-sensitive EC-9706 cells	PD-L1	↑ P-gp expression ↑ Resistance to paclitaxel	[164]
Paclitaxel-resistant CNE1 cells	Drug-sensitive CNE1 cells	DDX53	↑ P-gp expression ↑ Resistance to paclitaxel	[167]
Gefitinib-resistant PC9 and H1975 cells	Drug-sensitive PC9 cells	FTO	↑ BCRP and ABCC10 expression ↑ Resistance to gefitinib	[168]

Abbreviations: 5-FU: 5-fluorouracil; 22Rv1: prostate cancer cells; AML: acute myeloid leukemia; A549: non-small cell lung cancer cells; A2780: ovarian cancer cells; Bel7402: hepatocellular carcinoma cells; CCRF-CEM and CEM: acute lymphoblastic leukemia cells; CNE1: nasopharyngeal carcinoma cells; DDX53: DEAD-box helicase 53; DNAJB8: DNA J heat shock protein family (Hsp40) member B8; DU145: prostate cancer cells; EC-9706: esophageal squamous cell carcinoma cells; Eca109: esophageal carcinoma cells; EV: Extracellular vesicle; FTO: fat mass and obesity-associated protein; H1299: non-small cell lung cancer cells; H1975: lung adenocarcinoma cells; HepG2: hepatocellular carcinoma cells; HGC27: gastric cancer cells; hsa-circ: human circular RNA; HSC-5: cutaneous squamous cell carcinoma cells; Huh7: hepatocellular carcinoma cells; lnc: long noncoding RNA; KATOIII: gastric cancer cells; KB: human oral epidermoid carcinoma cells; LS174T: human colorectal cancer cells; MG-63: osteosarcoma cells; MCF7: breast cancer cells; MDA-MB-231: triple-negative breast cancer cells; miR: microRNA; MGC-803: gastric cancer cells; MPC-11: multiple myeloma cells; MRP1: multidrug resistance-associated protein 1; MSC: mesenchymal stem cell; Nrf2: nuclear factor erythroid 2-related factor 2; PC9: lung adenocarcinoma cells; PD-L1: programmed death ligand 1; P-gp: P-glycoprotein; SGC-7901: gastric cancer cells; SKBR3: breast cancer cells; SKOV3 and SKOV3-ip1: ovarian cancer cells; SMMC-7721: hepatocellular carcinoma cells; SW480: primary colorectal cancer cells; SW620: metastatic colorectal cancer cells; TrpC5: transient receptor potential channel 5; U937: human acute myeloid leukemia cells; UCH-L1: ubiquitin carboxy-terminal hydrolase L1. ↑: up-regulation or increase; ↓: down-regulation; ?: relevant EV cargo is not known.

**Table 2:** Inhibition of drug resistance through EV-mediated cell–cell communication. Described are cases where EVs released by donor cells led to the inhibition of multidrug resistance upon addition to target cells. The relevant EV cargo, at least partially responsible for the effects, if known, is also indicated.

Donor Cell	Target Cell	Relevant EV Cargo	Effect on the Target Cell	Reference
P-gp-overexpressing CEM cells	MRP1-overexpressing CEM cells	miR-326	↓ MRP1 mRNA and protein expression	[133]
Drug-sensitive SGC-7901 and MGC-803 cells	5-FU/cisplatin-resistant SGC-7901 cells	miR-107	↓ P-gp expression ↑ Sensitivity to 5-FU and cisplatin	[134]
Parental MG-63 cells (treated with luteolin)	Doxorubicin-resistant MG-63 cells	miR-384	↓ P-gp expression ↑ Sensitivity to doxorubicin	[135]
Drug-sensitive MCF7 cells (treated with β-elemene)	Doxorubicin- and docetaxel-resistant MCF7 cells	↑ miR-34 and ↓ miR-452	↓ P-gp expression	[136]
hCECs (nontumoral cells)	HepG2 and Hep3B cells	miR-214	↓ P-gp expression ↑ Sensitivity to oxaliplatin and sorafenib	[137]
MSCs	U87 and T98G cells	anti-miR-9	↓ P-gp expression	[138]
Oxaliplatin-resistant SW480 and HCT116 cells	Drug-sensitive SW480 and HCT116 cells	circ_FBXW7	↑ Sensitivity to oxaliplatin ↓ miR-18b-5p and MRP1 expression	[155]

Abbreviations: 5-FU: 5-fluorouracil; CEM: acute lymphoblastic leukemia cells; circ: circular RNA; HCT116: colorectal cancer cells; hCEC: human cerebral endothelial cells; Hep3B and HepG2: hepatocellular carcinoma cells; MCF7: breast cancer cells; miR: microRNA; MGC-803: gastric cancer cells; MRP1: multidrug resistance-associated protein 1; P-gp: P-glycoprotein; SGC-7901: gastric cancer cells; SW480: primary colorectal cancer cells; T98G and U87: glioblastoma multiforme cells; MSCs: mesenchymal stem cells. ↑: up-regulation or increase; ↓: down-regulation.

## Data Availability

Not applicable.
